# Peptides as Pharmacological Carriers to the Brain: Promises, Shortcomings and Challenges

**DOI:** 10.1021/acs.molpharmaceut.2c00523

**Published:** 2022-09-29

**Authors:** Sofia Parrasia, Ildikò Szabò, Mario Zoratti, Lucia Biasutto

**Affiliations:** † Department of Biology, University of Padova, Viale G. Colombo 3, 35131 Padova, Italy; ‡ CNR Neuroscience Institute, Viale G. Colombo 3, 35131 Padova, Italy; § Department of Biomedical Sciences, University of Padova, Viale G. Colombo 3, 35131 Padova, Italy

**Keywords:** peptides, blood-brain-barrier, receptor-mediated transcytosis, cell-penetrating peptides, drug delivery

## Abstract

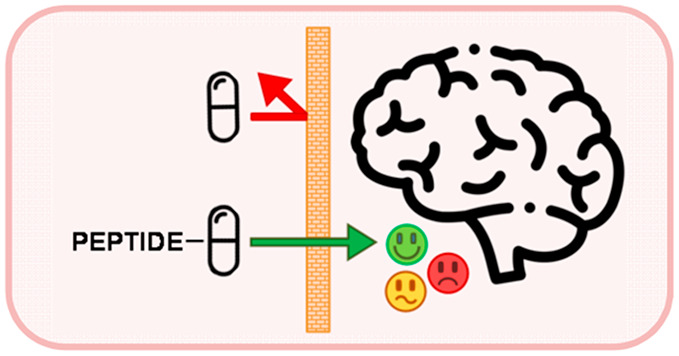

Central nervous system (CNS) diseases are among the most difficult to treat, mainly because the vast majority of the drugs fail to cross the blood-brain barrier (BBB) or to reach the brain at concentrations adequate to exert a pharmacological activity. The obstacle posed by the BBB has led to the in-depth study of strategies allowing the brain delivery of CNS-active drugs. Among the most promising strategies is the use of peptides addressed to the BBB. Peptides are versatile molecules that can be used to decorate nanoparticles or can be conjugated to drugs, with either a stable link or as pro-drugs. They have been used to deliver to the brain both small molecules and proteins, with applications in diverse therapeutic areas such as brain cancers, neurodegenerative diseases and imaging. Peptides can be generally classified as receptor-targeted, recognizing membrane proteins expressed by the BBB microvessels (e.g., Angiopep2, CDX, and iRGD), “cell-penetrating peptides” (CPPs; e.g. TAT_47–57_, SynB1/3, and Penetratin), undergoing transcytosis through unspecific mechanisms, or those exploiting a mixed approach. The advantages of peptides have been extensively pointed out, but so far few studies have focused on the potential negative aspects. Indeed, despite having a generally good safety profile, some peptide conjugates may display toxicological characteristics distinct from those of the peptide itself, causing for instance antigenicity, cardiovascular alterations or hemolysis. Other shortcomings are the often brief lifetime *in vivo*, caused by the presence of peptidases, the vulnerability to endosomal/lysosomal degradation, and the frequently still insufficient attainable increase of brain drug levels, which remain below the therapeutically useful concentrations. The aim of this review is to analyze not only the successful and promising aspects of the use of peptides in brain targeting but also the problems posed by this strategy for drug delivery.

## 1 Introduction: The Marvelous World of Peptides

The astounding success of life on Earth is largely due to the versatility provided by the mathematical “rule of product” incorporated into the polymeric fabric of living matter. The 20 standard amino acids can in principle be combined to produce 20^*N*^ sequences, where *N* is the number of monomers in the (linear) chain. Thus, nature can use evolution to pick the molecule most suitable for any given biochemical task, selecting among 8000 possible tripeptides, 160 000 tetrapeptides, 200 billion decapeptides, and so forth. Relatively short peptides, of up to, say, 30 monomers, seldom act as enzymes, but they have plenty of other functions. They can be selectively toxic for microorganisms and thus constitute a first line of defense against infections by cellular organisms (host defense peptides) and viruses, inspiring man-made or “borrowed” peptide antibiotics. Vice versa, powerful peptide toxins are produced by many microorganisms and animals, and also find or hope to find much pharmaceutical use. Peptides can be immunomodulatory, with an impact on inflammation and cancer. A list of those acting as hormones would be long. They offer hope as anticancer vaccines or as “direct” chemotherapeutics. Just as relatively short amino acidic sequences may have egregious physiological effects, relatively short polypeptide domains are often directly responsible for specific features of a protein’s activity or behavior. This offers a window of opportunity for pharmacologists, who can discover or engineer appropriate peptides to inhibit, activate, compete, direct.

To mention just one currently relevant example of such an application, interfering with protein–protein interaction, peptides are being developed that compete with the binding of the SARS-CoV-2 virus Spike protein to its receptors, the major one being angiotensin-converting enzyme 2 (ACE-2) (for reviews, see refs (1−3)). A brief overview of current pharmaceutical applications of peptide-drug conjugates can be found in refs (4) and (5). The perspectives of food-derived peptides are summarized in ref (6).

Besides the versatility of peptides, an advantage for researchers is, generally speaking, the ease of their synthesis by standard solid-phase procedures and of their characterization by established methods. Another is the possibility of screening large random libraries selecting effective peptides thanks to phage, yeast, bacterial, and other forms of display/biopanning technology.^7^ The isolated sequences can be produced (and modified/adapted) and used to build drug conjugates or to decorate nanovehicles for selective delivery.^8^ Phage display can be used for biopanning *in vivo*: phage libraries can for example be infused into the circulatory system, and the phages remaining most tenaciously associated with a given organ/compartment/cell type (e.g., the epithelial surface of the BBB) can be isolated through multiple rounds of selection (for a review, see ref (9)).

Besides discovery via phage display, this example illustrates the use of peptides to target vascular “receptors” for pharmacological purposes (i.e., either to alter the functionality of the target protein or to use it as a docking site for the delivery of a “cargo” that may be a small molecule or a nanovehicle). For efficient cargo delivery, obviously the receptor ought to be strongly expressed on the luminal surface of the targeted vasculature and ought to have a fast transcytosis or endocytosis turnover (see below).

The numbers of such peptides, their known receptors, and clinical trials testing them mostly for oncological and cardiovascular applications run into the dozens.^10−13^ Among the most popular target-recognizing sequences are the RGD (or KGD) motif, which homes to integrins and the NGR triplet, which recognizes instead CD13, an aminopeptidase overexpressed by tumor vascular cells.^14,15^ These motifs may exhibit a higher affinity for their targets when presented within conformationally constrained, cyclized, peptides.^16^

We focus now on the use of targeting peptides to aid the delivery of small-molecule drugs (or other peptides) and nanovehicles to the brain vasculature and parenchyma.

In this paper the amino acid sequences of peptides are written following the usual convention (i.e., with the N-terminal at left). Amino acids with the natural (l) configuration are denoted by their uppercase one-letter code, while unnatural (d) enantiomers are indicated by the use of the lower case. A lower-case “d” preceding a peptide’s nickname (label/abbreviation) indicates that the peptide is formed by d-amino acids (e.g., dA7R), while a “c” preceding peptide’s name or sequence indicates a cyclic peptide. For readability, in the main text peptides are often mentioned by their abbreviation, without giving the amino acid sequence, which can however be found in the tables.

## 2 The Blood-Brain Barrier (BBB)

### 2.1 BBB Function and Structure

The BBB, discovered by Paul Ehrlich in 1885, is the interface separating circulating blood from the brain parenchyma in the central nervous system (CNS) (for an overview of human cerebral vasculature, see ref (17)). Far from being a fixed structure, it changes in time and space.^18−20^ The main functions of the BBB are the protection of the brain from external agents, either chemical or biological, that could damage it, and the maintenance of the correct homeostasis for optimal neuronal function.^18^

**Figure 1:**
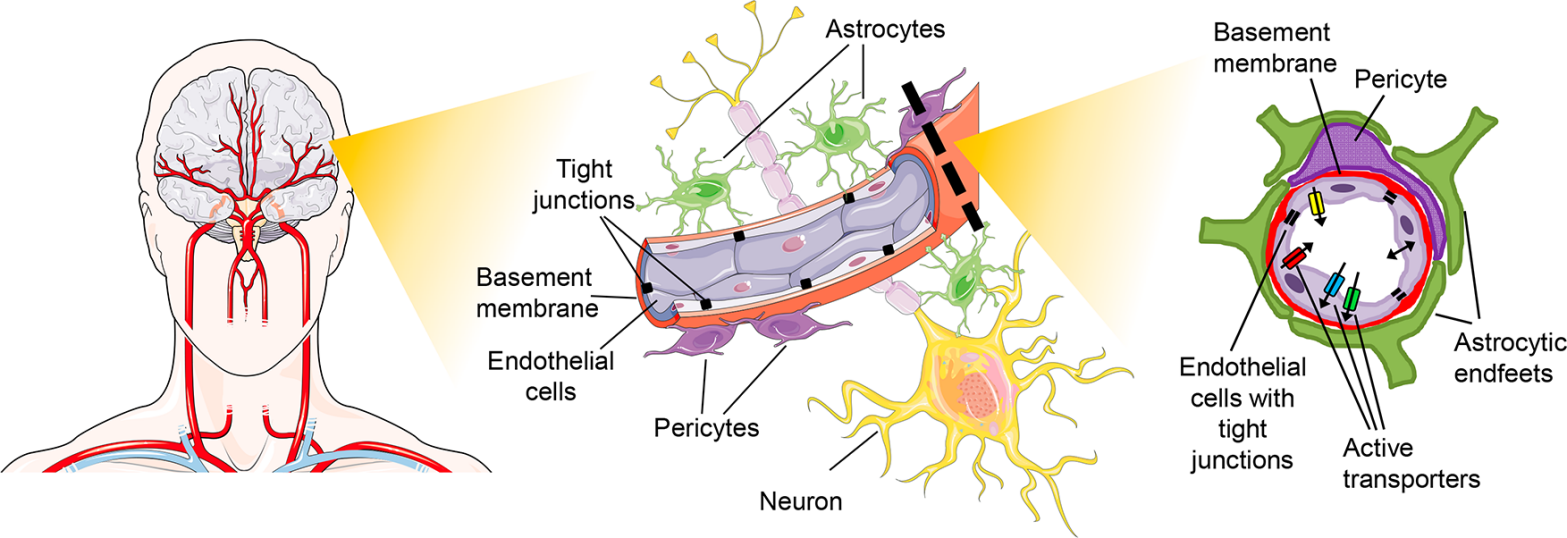
Overview of the multicellular structure of the BBB.

The BBB is a multicellular structure, with the participation of pericytes, astrocytes, microglia, neurons, and a basal membrane, which helps the anchoring of the cells (Figure 1). Astrocytes are important for the modulation of the expression of transporters and receptors and for fine-tuning the tight junctions (TJs; see below) and efflux pumps. Pericytes exert a major role in the modulation of the trans-endothelial resistance, of the rate of transcytosis, and of the expression of efflux pumps.

**Figure 2:**
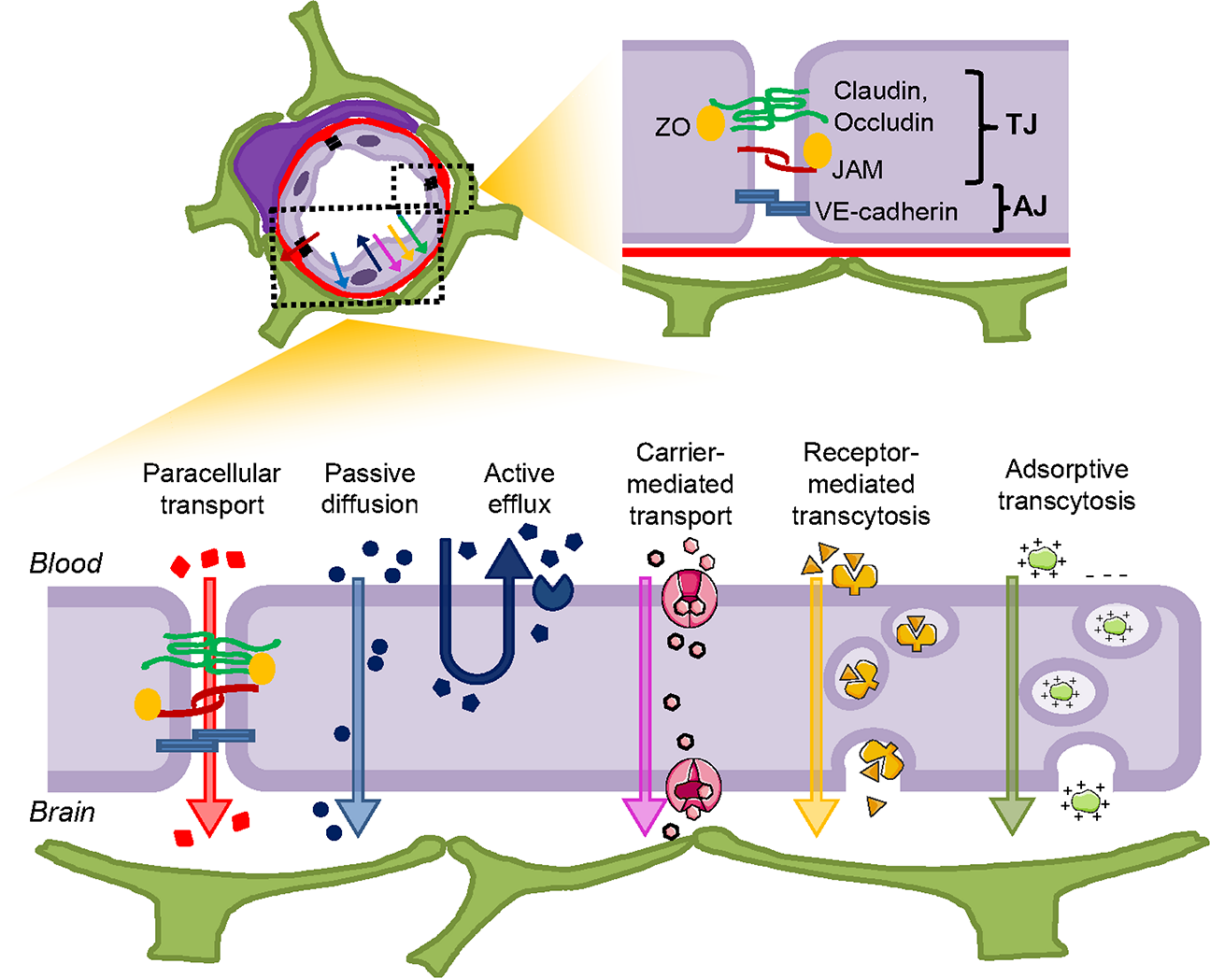
Junctional complexes of the BBB and permeation pathways across it.

The functionality of the BBB is ensured by the presence of two main junctional complexes, namely, tight junctions (TJs) and adherens junctions (AJs), connecting the endothelial cells of the brain capillaries that selectively regulate the influx and efflux of substances through the paracellular pathway^21^ (Figure 2).

AJs play a role in the maintenance of cellular polarity and in the stability and survival of endothelial cells. They are located in the basal region of the lateral plasma membrane and are mainly built by vascular endothelial cadherin (VE-cadherin), which forms homophilic cell–cell junctions. The reciprocal interaction of cadherin building blocks is Ca^2+^-dependent but also needs the presence of catenins, which together with other proteins act as anchor molecules connecting cadherin to the actin cytoskeleton.

TJs are essential for the integrity of the BBB, especially for the maintenance of the trans-endothelial electrical resistance. They are formed by up to 40 different proteins such as claudins (CLDN), occludin (OCLN), zonula occludens (ZO), junctional adhesion molecules (JAM), and others. CLDNs are characterized by four transmembrane domains with two extracellular loops and are fundamental in the formation of TJ strands. CLDN-5 is the major claudin of the BBB. The composition of CLDNs determines the molecular weight (MW) of molecules that can cross the junction. OCLNs are also involved in the MW cutoff for crossing the BBB and, in particular, they are very selective for low-MW molecules. Another important TJ protein family is that of the JAM, which are type I single-transmembrane proteins. Within this family, JAM-A is highly expressed in the brain and limits the passage of molecules with MW higher than 4 kDa by forming close membrane appositions.^22^ The ZO family connects TJs transmembrane proteins to the actin cytoskeleton and stabilizes TJ strands. The presence of ZO-1 and -2 is fundamental for the formation of TJs.^22^

While the exchange of small as well as larger molecules is essential to support the high metabolic demands of the brain, the structure of the BBB makes the delivery of drugs to the brain difficult. The problem of overcoming it to deliver psychotropic agents or drugs against CNS cancers, neurodegeneration, neuroinflammatory states, autoimmune disorders, and so on has vexed generations of researchers and physicians.^23−30^ It is estimated that only 2% of “small” molecules can cross the BBB, regardless of their beneficial or noxious effects.^24^

Generally speaking, the diffusion through the BBB can be achieved by para- or transcellular pathways (see below, sections 2.2 and 2.3; Figure 2).

### 2.2 BBB Permeation: Paracellular Transport

In the healthy brain, the passage of substances through the intercellular space between endothelial cells of the BBB (i.e., the paracellular transport) is dramatically restricted because of the presence of TJs and AJs (Figure 2). Pathological conditions such as neuroinflammatory states, neurodegenerative diseases, or cerebral cancers may be associated with a loss or decrease of BBB integrity.^31^ Alterations induced for drug delivery purposes obviously need to be transient. Many efforts have been made to alter the permeability of the BBB using broadly acting approaches. These include transiently loosening the TJs with vasoactive compounds such as histamine or agonists of the A_2A_R adenosine receptor^32^ (the latter also downregulate the expression of efflux “pumps”).^33^ Osmotic agents (e.g., mannitol),^34^ ultrasound,^35^ X-rays,^36^ electromagnetic fields,^37^ and increasing the temperature in a focused manner (e.g., with microwave beams)^38^ have also been used.

TJ tightness is regulated by phosphorylation and dephosphorylation of essentially all participating proteins by several kinases, in a complex and not fully understood manner.^39,40^ For instance, phosphorylation at the C-terminus of CLDNs by PCKβ counteracts their interaction with ZO-1. However, phosphorylation of OCLN and ZO-1 is essential for the integrity of the BBB, but additional phosphorylation can lead to barrier disruption.^41^ A localized, reversible, and specific modulation of kinase and/or phosphatase activity might thus be a way to help drugs enter the brain. Little research in this direction seems to have been conducted so far.^38,42^

Molecular-size-specific approaches for the modulation of BBB can be achieved by the use of RNA interference;^38,42^ for example, siRNA administration was used to knock-down CLDN-5 and thus to allow the delivery of molecules up to 1 kDa to the brain.

Extracellular vesicles (EVs) are able to cross the BBB in either direction.^43,44^ Even though the exact transport pathways have not yet been fully clarified, it is interesting that in a zebrafish model EVs and exosomes (EXOs)^30^ holding miRNA miR-132 can modulate the expression of VE-cadherin. Interference with the expression of neuronal miR-132 or with the secretion of miR-132 containing EXOs leads to an increase in the BBB permeability.^45^

The controllers of junctional tightness can also be targeted by other means.^38,42^ For example, claudin and occludin can be engaged by fragments of bacterial toxins or antibodies.^46^ Peptides directed against components of the cell–cell interfaces have been used in several studies.^38,42^ Anti-VE-cadherin mAbs and peptides have been used to strongly modulate BBB permeability.^47^ Bocsik and co-workers identified a set of short peptides recognizing components of intercellular junctions which induced a marked decrease of the trans-endothelial electrical resistance (TEER) and an increased permeability of a ternary cocolture BBB *in vitro* model. The authors proposed that these peptides might represent suitable excipients to improve drug absorption.^48^ The concentrations applied in their experiments were however relatively high, ranging from 10 μM to 2 mM. In analogous work, claudin peptidomimetics, binding with nanomolar-range affinity to extracellular loop 1 of CLDN-5, were able to transiently loosen the junctions of bEND.3 cells, a mouse BBB model, and of a more complex model formed by filter-grown primary rat brain endothelial cells cocultured with pericytes and glial cells. The effect was associated with redistribution of CLDN-5 from the membrane to the cytosol and with morphological changes of the cells. The mRNAs of CLDN-5, ZO-1, and occludin were reduced. All effects could be reversed by washing off the agent. *In vivo* injection of 3.5 μmol/kg of body weight (bw) of C5C2 (the best performer: a 29 aa peptide based on a segment of the first extracellular domain of CLDN-5) determined an increase in the amount of trackers reaching the brain.^49^ Similar results were obtained with another 29 aa peptide (C1C2) targeting CLDN-1, applied to models of the peripheral nerve–blood barrier.^50^

### 2.3 BBB Permeation: Transcellular Transport

#### 2.3.1 Passive Diffusion

Transmembrane diffusion is a nonsaturable process that mostly depends on physicochemical characteristics of the molecules such as molecular weight and lipid solubility (Figure 2). The ideal MW should not exceed 400 Da. Already some 50 years ago it was pointed out that a MW increase of 150 Da is enough to cause a 100-fold decrease in BBB permeation.^51^ The characteristics that collectively should be present in a drug addressed to the CNS are summarized by the well-known “Lipinski’s rule of five”: Besides a reasonably low MW, they include a limit on hydrogen bonds (<6), a clear lipophilicity (Log*P* > 2), the absence of free rotatable bonds, and a polar surface area <60 Å.^52,53^ Methods have been proposed to estimate *a priori* the “CNS druggability” of a given drug on the basis of its composition and structure.^54,55^ Thus, while some small molecules such as some lipid-soluble compounds can cross the BBB by passive diffusion, molecules with higher molecular weight, bearing electrical charges, or with marked polarity or hydrophobicity need to exploit facilitated transport.

#### 2.3.2 Carrier-Mediated Transport

The BBB expresses in a development-dependent manner^56^ various transporters in order to satisfy the energetic and nutritional demands of the brain. Among those functionally devoted to influx are carriers for l-type amino acids (LAT1, which can also transport drugs such as l-DOPA, gabapentin, or mephalan due to structural similarities with the endogenous ligands),^57^ glucose (GLUT1),^58^ monocarboxylates (MCT1),^59^ cationic amino acids (CAT1),^60^ choline (ChT),^61^ and possibly organic cation transporters (OCT/OCTN)^62^ and sodium-coupled glucose transporters.^63^ These carriers can be exploited to facilitate the transport of appropriate prodrugs across the BBB^57,64,65^ (Figure 2).

#### 2.3.3 Efflux Transport

Efflux transporters are represented by various ATP-driven drug “pumps”, including P-glycoprotein (P-gp), breast cancer resistance protein (Bcrp), and the multidrug resistance-related proteins (Mrp1, 2, 4, and 5). These contribute to limiting the entry of drugs and toxins into the brain (Figure 2). They are expressed on the luminal side of brain capillaries^66^ and are regulated by various mechanisms, including WNT signaling.^67^ Inhibition of efflux pumps or of their expression is one possible approach to increasing the net influx of drugs into the brain parenchyma (e.g., refs (33), (68), and (69)). Coupling the drug to a BBB-penetrating peptide may allow it to avoid PgP action.^70,71^

### 2.4 BBB Permeation: Transcytosis

#### 2.4.1 Receptor-Mediated Transcytosis

This family of processes is normally used for the uptake of relatively bulky molecules or complexes. Receptor-mediated trancytosis (RMT), which exploits the presence of specific receptors at the BBB, is highly specific and provides the uptake of the receptor ligand from the luminal side of the endothelial cells to the brain (Figure 2). RMT is a complex and still incompletely understood process involving clathrin- and caveolin-coated vesicles, the delivery of ligands to the basal membrane avoiding the lysosomal degradation pathway, and the recycling of receptors.^72−74^ Transcytosis is lower in the BBB than in other endothelia,^75^ due to regulation by Major facilitator superfamily domain containing 2a (Mfsd2a)^76^ and possibly other controllers, and to the influence of pericytes.^77^ A major role in traffic control is played by Rab small GTPases. Since multiple processes are possible downstream of ligand–receptor interaction, RMT appears to be sensitive to factors such as the binding affinity^78^ and hence, presumably, to details of the ligand structure (e.g., the incorporation of tags or linkers). A better understanding of the regulation of transcytosis may provide the key to efficient drug delivery to the brain.

#### 2.4.2 Adsorptive Transcytosis

Receptor-independent (or “adsorptive”) transcytosis does not involve interactions with specific membrane proteic receptors, but rather, it is thought, with membrane-associated negative charges, such as sulfate or phosphate groups in glycoproteins or the phospholipid head groups of the lipid bilayer (Figure 2). Uptake then takes place via processes such as pinocytosis, lipid-raft-mediated internalization, endocytosis.^79^ Adsorptive transcytosis is characteristic of nanovehicles decorated with cell-penetrating peptides (CPPs; see below).

These pathways are discussed further below, in connection with peptide-mediated brain delivery. A variety of nanovehicles have been engineered to favor receptor-dependent or receptor-independent transcytosis of the transported drug.^80,81^

## 3 Peptides as Pharmacological Carriers to the Brain: Promising Aspects

Peptides recognizing specific components or features of the CNS microvessel luminal surface represent a useful strategy to target the BBB and overcome it. Peptides are also used to build conjugates comprising the active principle, in many cases a peptide itself, linked stably or in prodrug fashion (e.g., refs (82−84)). They are, despite the limitations which we shall discuss, the most promising pharmacological tool available.^85,86^ A database containing an updated list of all the BBB-penetrating peptides studied so far has been recently built up by Raghava’s group (B3Pdb database: https://webs.iiitd.edu.in/raghava/b3pdb/).^87^

### 3.1 Receptor-Targeted Peptides

Various receptors expressed on the surface of brain microcapillaries have been investigated as potential brain parenchyma entry points by RMT.^88^ They prominently include the transferrin receptor (TfR),^89,90^ low-density lipoprotein family receptors (LDLR)^91−93^ including low-density lipoprotein receptor-related protein 1 (LRP1),^94^ the nicotinic acetylcholine receptor (nAchR)^95^ and the leptin receptor (leptin R).^96^

Table 1 presents a tabulation of literature reports concerning peptides targeting identified BBB receptors. Table 2 lists peptides discovered using phage display and thus also presumably recognizing still-unidentified BBB proteins.

**Table 1:**
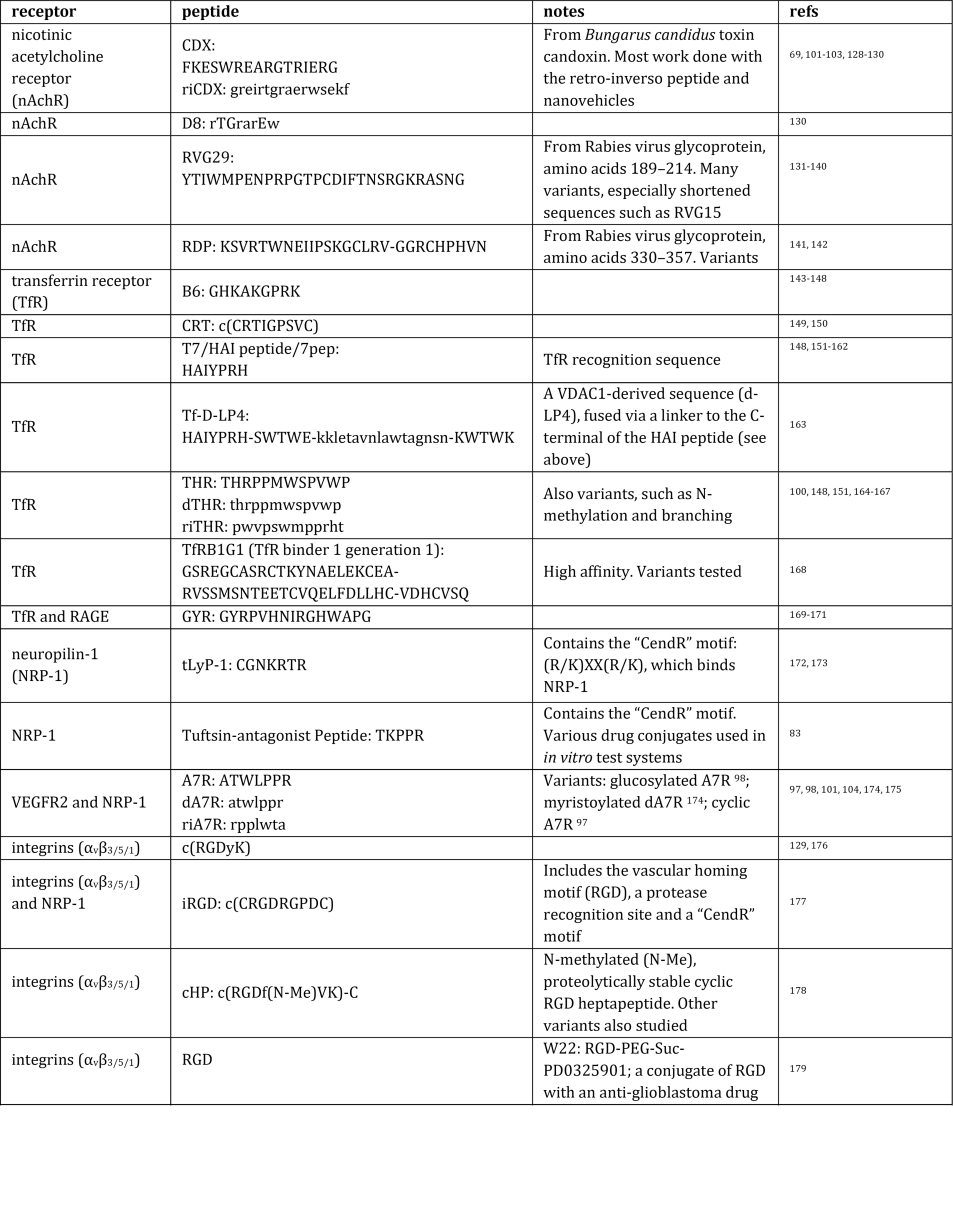

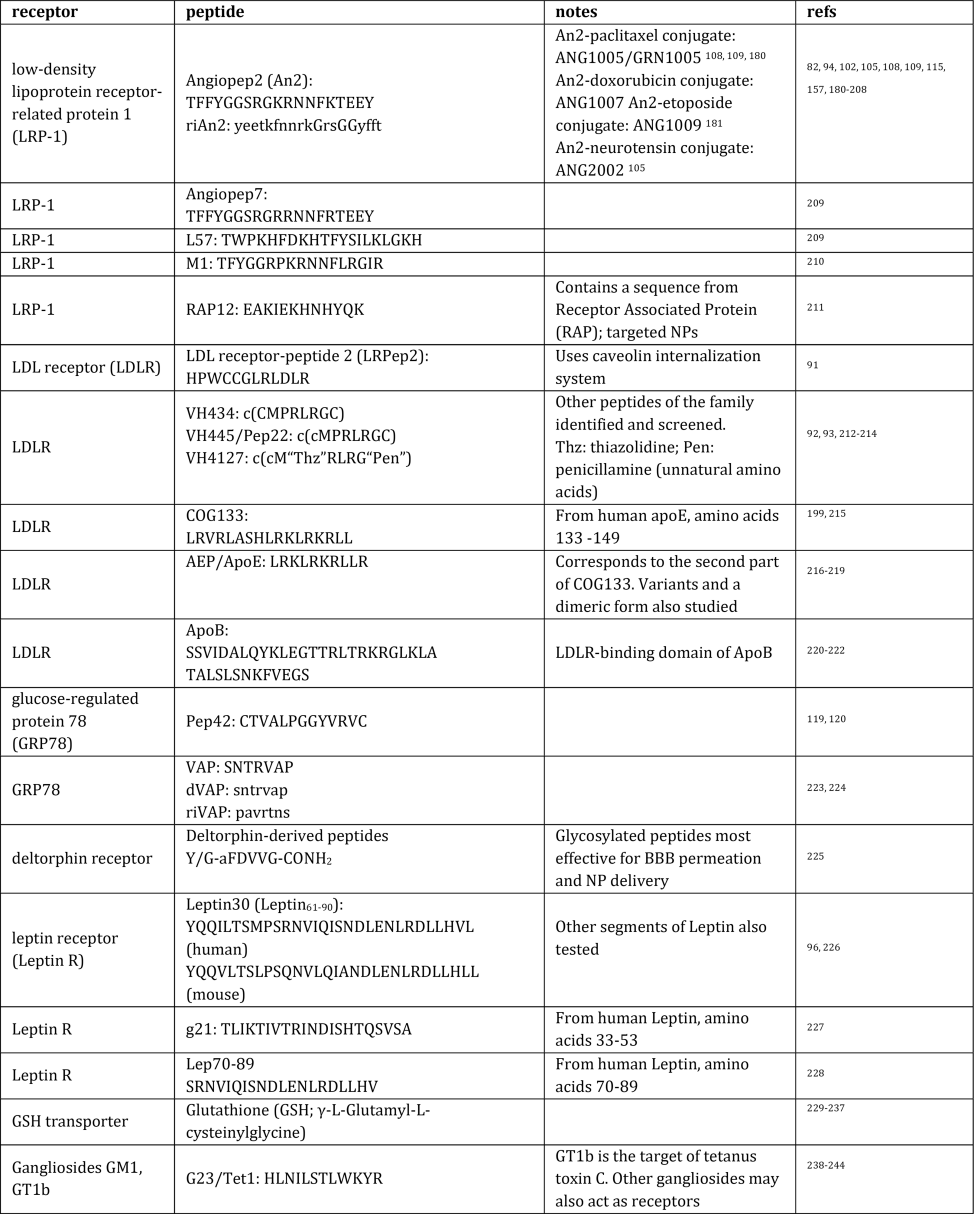

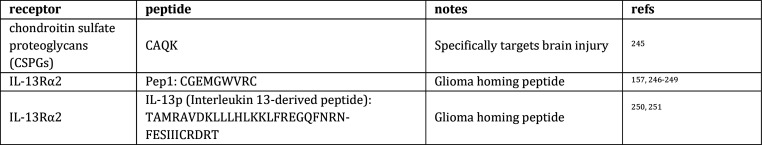
Receptor-Targeting Peptides^69,83,97−105,108,109,115,128−251^

**Table 2:** Peptides (Discovered by Phage Display) Presumably Targeting an Unidentified BBB Protein

peptide	notes	refs
YtGFLS(β-d-glucose)-CONH_2_	Glucosylated peptides are derived from enkephalin; GLUT-1 may be involved in BBB crossing.	(252)
glioma-homing peptide (gHo): NHQQQNPHQPPM	Fusion constructs with peptides pVEC, SynB3, andAn2 have been studied (see Table 4).	(253)
CAGALCY		(254,255)
PepC7: c(CTSTSAPYC)		(256)
GLHTSATNLYLH		(257)
VAARTGEIYVPW		(257)
SGV: SGVYKVAYDWQH	other sequences also evaluated; internalization by clathrin-coated pits	(258)
TPS: TPSYDTYAAELR	permeation of the blood–cerebrospinal fluid barrier	(259)
c(AC-SYTSSTM-CGGGS)	AC and CGGGS are flanking sequences used to cyclize; other similar sequences identified.	(260)
GLA: GLAHSFSDFARDFVA	adhesion to brain microvasculature	(169,170)
GYR: GYRPVHNIRGHWAPG		
brain-homing peptide (BH): CNAFTPDY	used for DNA delivery	(261)
CLEVSRKNC	other similar sequences identified; targets ischemic area of rat brain	(262)
miniAp-3: c(CKAPETALC)	brain-targeting peptide based on apamin; other variants also	(263,264)
TGN: TGNYKALHPHNG		(265−268)
EI-3: FSRPAFL	other less promising peptides identified; *in vitro* studies only	(269)
CLSSRLDAC	other brain-homing peptides also identified: CNSRLHLRC, CENWWGDVC, WRCVLREGPAGGCAWFNRHRL	(270)
TACL05: c(CSACPSHLTKMC)	other peptides also identified	(271)
RLSSVDSDLSGC	other peptides identified, including: LYVLHSRGLWGFKLAAALE, LGSVS, GFVRFRLSNTR	(272)
EI-3: FSRPAFL	several other peptides identified; internalized by medulloblastoma cell.	(269)
SLS: SLSHSPQ	cyclized form also tested	(273)
c(ACSLSHSPQ-CGGGS)		

Among the former, we may single out as an example A7R, identified via phage display, which is a specific ligand for VEGFR-2 and neuropilin-1 (NRP-1).^12^ By binding to one or the other of these two partnering receptors, A7R prevents their association and thus impacts angiogenesis. These receptors are highly expressed also in glioma cells, making A7R a candidate weapon against CNS cancers. It turned out however to be rapidly degraded by proteases and to be excluded by the BBB. The stability problem was approached by constructing a head-to-tail cyclized derivative, which retained much the same binding properties as the linear peptide.^97^ A glycosylated derivative, intended to exploit the GLUT-1 transporter abundant in brain microvessels, was reported not only to be more stable but also to traverse the BBB and to successfully deliver paclitaxel-loaded “nanodisks” to orthotopically implanted U87MG glioma cells after intravenous (i.v.) administration.^98^ A more widespread and effective approach to stabilization, used also with A7R, is to construct peptides with d-amino acids,^99^ which are not recognized by peptidases. These unnatural peptides may be built with the same amino acidic sequence of the natural parent peptide or with the reverse one (retro-inverso, ri) (for an example of the latter, see ref (100)).

Ying et al.^101^ used both dA7R and dCDX, a d-peptide ligand of nicotinic acetylcholine receptors (nAChRs) derived from candoxin and capable of passing the BBB,^102,103^ to decorate liposomes that functioned as hoped, overcoming the BBB to deliver their content of doxorubicin to glioma more efficiently than liposomes decorated with one or the other of the individual peptides.

Zhang and Lu coupled dA7R with another peptide, GICP, also identified via phage display, which binds to VAV3, a Rho-GTPase GEF highly expressed by glioma cells. The construct showed improved homing and BBB-crossing abilities.^104^

The angiopep (An) family of peptides targets instead LRP1.^94,105^ These were derived from the Kunitz protease-inhibitor domain (present also in secreted β-amyloid precursor protein) of aprotinin, a 6500 Da protease inhibitor that can cross the BBB.^105^ Several studies have upheld its ability to facilitate the cross-BBB delivery of “cargo”, including nanovehicles (see Table 1). Again, since LRP-1 is highly expressed in astrocytomas, especially glioblastomas,^106,107^ this vector is a potentially useful tool against CNS cancers. Indeed, an An2-paclitaxel conjugate (ANG1005)^108,109^ has reached the clinical trials stage.^110−112^ Our group has recently produced a conjugate of An2 with PAPTP, a triphenylphosphonium (TPP)-containing mitochondriotropic psoralene derivative which shows powerful anticancer activity^113^ but cannot cross the BBB.^114^ Conjugation to the peptide, a first for TPP-decorated molecules, allowed brain delivery.^115^

Transferrin receptors are abundant in brain capillary endothelial cells and in rapidly dividing cells and immature erythroid cells.^116^ They are however scarce in other vasculature and tissues, and this provides a built-in selectivity which has made this system a popular target for receptor-mediated delivery attempts^89,90^ (see Table 1).

A strategy waiting to be tested may involve Glucose-regulated protein 78 (GRP78, also called immunoglobulin heavy-chain binding protein or BiP), a heat shock protein with endoplasmic reticulum (ER) regulatory functions, expressed in the ER of the vast majority of cells. GRP78 is also expressed on the surface of cancer cells,^117,118^ including glioma and angiogenic epithelial cells. This overexpression has been linked to malignant behavior, including drug resistance. Thus, GRP78 has been investigated for cancer therapy, and a cyclic 13-mer peptide called Pep42 has been designed to selectively target it.^119,120^ Recent findings have shown that GRP78 is found on the cell surface of brain microvascular endothelial cells, and that autoantibodies against GRP78 are associated with CLDN-5 downregulation and BBB loosening in neuromyelitis optica^121^ and systemic lupus erythematosus.^122^ In rats treated with the mitochondrial toxin 3-nitropropionic acid, vascular GRP78 expression was spatially and temporally correlated with BBB leakage.^123^ Collectively, these observations suggest the possibility to use GRP78-specific peptides to reversibly loosen and bypass the BBB.

Parenthetically, GRP78 is a candidate receptor for the spike protein of SARS-CoV-2,^124,125^ for the ZIKV protein of Zika virus,^126^ and for glycoproteins GP1 and GP2 of Ebola virus.^127^

### 3.2 Cell-Penetrating Peptides

An alternative to receptor-targeting peptides is offered by so-called “cell-penetrating peptides” (CPPs), a large catalogue (about 1855 unique sequences are currently listed in CPPsite 2.0 database)^274,275^ of short chains that generally speaking can pass the membrane barrier thanks to their properties rather than specific interactions with proteins.^276−278^ Some efficiently permeate the BBB (Table 3), and they can be a useful tool for the delivery to subcellular compartments as well.^279,280^

**Table 3:** Peptides (CPPs) Facilitating Receptor-Independent BBB Transcytosis

peptide	notes	refs
TAT_47–57_: YGRKKRRQRRR	from HIV-1 TAT; variants depending on sequence stretch chosen; various cargos attached.	(84,297−315)
D3: rprtrlhthrnr	all-d peptide with a few homologies to TAT	(316−319)
penetratin_43–58_: RQIKIWFQNRRMKWKK	from *Drosophila* antennapedia homeodomain; several variants (e.g., dodeca-penetratin: RQIKIWFRKWKK)^320^	(136,320−325)
d-penetratin: rqikiwfqnrrmkwkk		
vascular endothelial-cadherin derived peptide (pVEC): LLIILRRRIRKQAHAHSK		(253,297,326,327)
transportan 10 (TP10): AGYLLGKINLKALAALAKKIL	abbreviated form of transportan (a combination of the N-termini of galanin, a porcine neuropeptide, and mastoparan, a pore former in wasp venom); transportan 10–2 differs by substitution of the second A by a P.	(297,328,329)
SynB1: RGGRLSYSRRRFSTSTGR	from protegrin, a natural antimicrobial peptide; various drug conjugates	(321,330−333)
SynB3: RRLSYSRRRFrrlsysrrrf	truncated derivative of SynB1; various drug conjugates	(70,83,297,330,332,334−337)
G7: GFtGFLS[*O*-β-d-glucose]	from the opioid peptide MMP-2200	(338−343)
deltorphin-derived peptides: GaFDVVG; GaFN(β-GlcNAc-OH)DVVG	drive NPs across BBB	(225)
PepH3: AGILKRW	α-helical domain of the dengue virus type-2 capsid protein; variants also	(344,345)
dPepH3: agilkrw		
PepNeg: SGTQEEY	negatively charged permeating peptide (the only case as far as we know)	(281)
*N*-MePhe-rich peptides (e.g., *N*-MePhe-(N-MePhe)_3_-CONH_2_)	short *N*-methyl-phenylalanine (*N*-MePhe) sequences coupled to small molecules; passive diffusion	(346,347)
(PhPro)_4_	phenyl-proline tetrapeptides; passive diffusion; improved solubility versus *N*-MeF peptides (see above); instances of enantiomeric selectivity in permeation	(348)
WSW/PhrCACET1: SYPGWSW	quorum-sensing peptide from *Clostridum acetobutylicum*; other peptides also investigated	(349,350)
riWSW: wswgpys		
NP2: KIKKVKKKGRK	from human novel LZAP-binding protein (NLBP), amino acids 444–454.; dimer of NP2 actually used	(315,351,352)
dimeric NP2: KIKKVKKKGRKGSKIKKVKKKGRK		
cytoplasmic transduction peptide (CTP): YGRRARRRRRR		(353,354)
LIMK2 NoLS peptide (LNP): KKRTLRKDRKKRC	nucleolar translocation signal sequence (NoLS) of LIM kinase 2 (LIMK2)	(355)
R7	poly-arginine peptide; variations (e.g., myristoylation)	(356)
R8	poly-arginine peptide	(310)
R11	poly-arginine peptide	(357)
R-rich peptide: (RXRRBR)_2_XB	X: any amino acid	(358)
	B: D/N	

They typically contain a high proportion of positively charged (basic) amino acids (cationic CPPs) or alternating patterns of charged and hydrophobic amino acids (amphipatic CPPs) (for an exception, see ref (281)). They can be variously classified depending on their origin, configuration (e.g., linear vs cyclic), physicochemical properties (e.g., charge, hydrophobicity, and length), and presumed mode of entry into cells (“direct translocation” and endocytosis). Endocytosis can proceed via pinocytosis and/or clathrin- and/or caveolin-mediated uptake, followed by escape from the endocytic pathway, which often is an important problem.^282,283^ As exemplified by two of the most exploited peptides, HIV-1 trans-activating protein-derived peptide (TAT) and penetratin,^284^ this sort of mechanism is used by many peptides derived from pathogens. Interaction with negatively charged cell surface molecules, such as proteoglycans/glucosaminoglycans (e.g., refs (285−288)) is believed to be an important early step during peptide uptake.

Direct, or energy-independent, translocation is envisioned to take place through one or the other of at least three mechanisms: formation of an oligomeric pore in the membrane (barrel stave model); adhesion to the phospholipidic cell surface, followed by a “disorderly” penetration and membrane alterations (on which see, e.g., discussion in ref (289)) (“carpet” model); and formation of inverted micelles at the cell membrane, which would then enter the cytoplasm. Processes of this sort are potentially dangerous for cell integrity. Indeed, membrane-lytic processes are the main mechanism of action of antimicrobial peptides, which have physicochemical properties similar to those of CPPs.^290^ Cationic CPPs can not only change the organization of membrane lipids, but also their composition. Specifically, Verdurmen and colleagues have shown that at high concentrations a contribution to the entry of cationic CPPs (they used oligoarginine) may be provided by a CPP-induced movement of acid sphingomyelinase from lysosomes to the outer leaflet of the cellular membrane, where the enzyme proceeds to generate ceramide, which facilitates peptide entry.^291,292^ CPPs can also induce various other cellular responses (as reviewed in ref (293)).

Given these multiple and complex features, it is unsurprising that the mechanistic details of cell entry may depend on the exact peptide sequence,^294^ the specific “cargo” attached to it,^295^ and/or the concentration of peptide or peptide-comprising construct.^277^

Besides the endosomal escape problem, which can impede the intracellular delivery of endocytosed agents because of their trapping in endosomes/lysosomes, a drawback of CPPs is that since they do not depend on the presence of a specific “receptor”, generally they are not very selective and tend to interact with all membranes (although, besides surface charge, lipid composition and membrane tension play a part). Nonetheless, a few are viewed as an effective instrument to overcome the BBB, generally with the intent of attacking CNS cancers such as glioma.^296,297^ These are tabulated in Table 3 and prominently include TAT (the first CPP to be discovered) and penetratin (derived from the Antennapedia protein homeodomain).

### 3.3 Combined Approaches

The major strategy fielded to counter lack of specificity is to combine a CPP and a specificity-conferring moiety (e.g., another peptide) on the surface of a nanovehicle^296,359^ (Table 4).

**Table 4:**
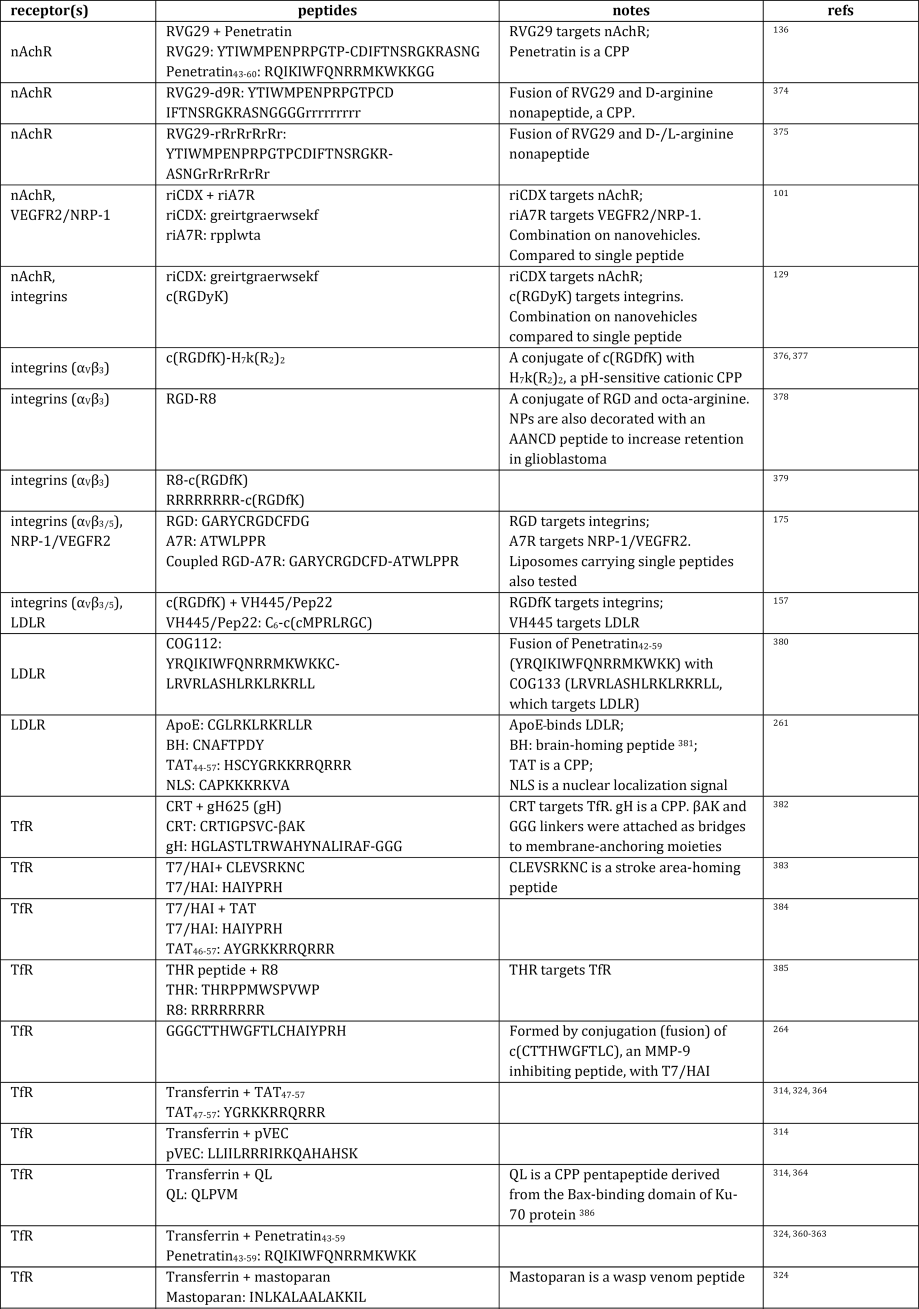

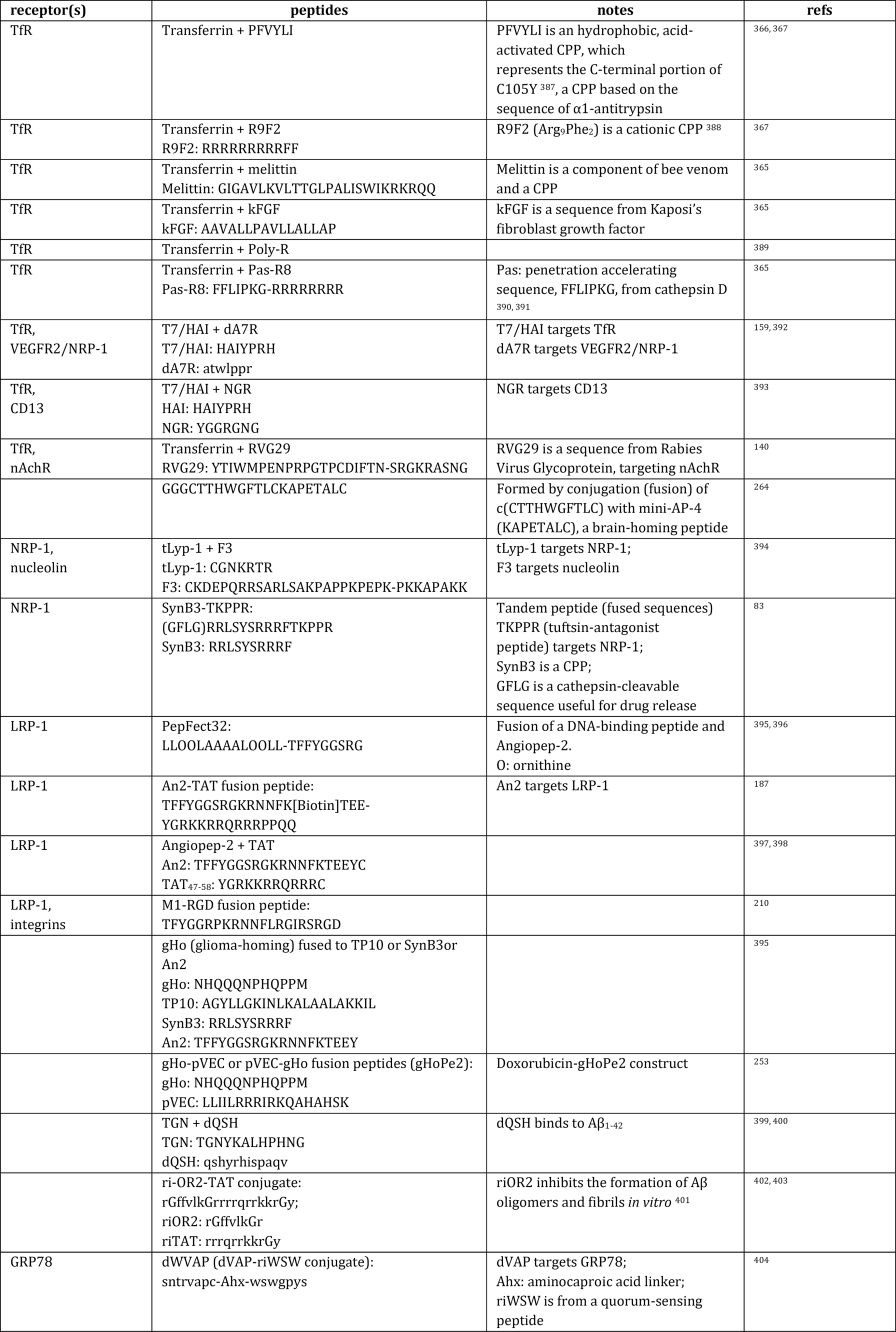

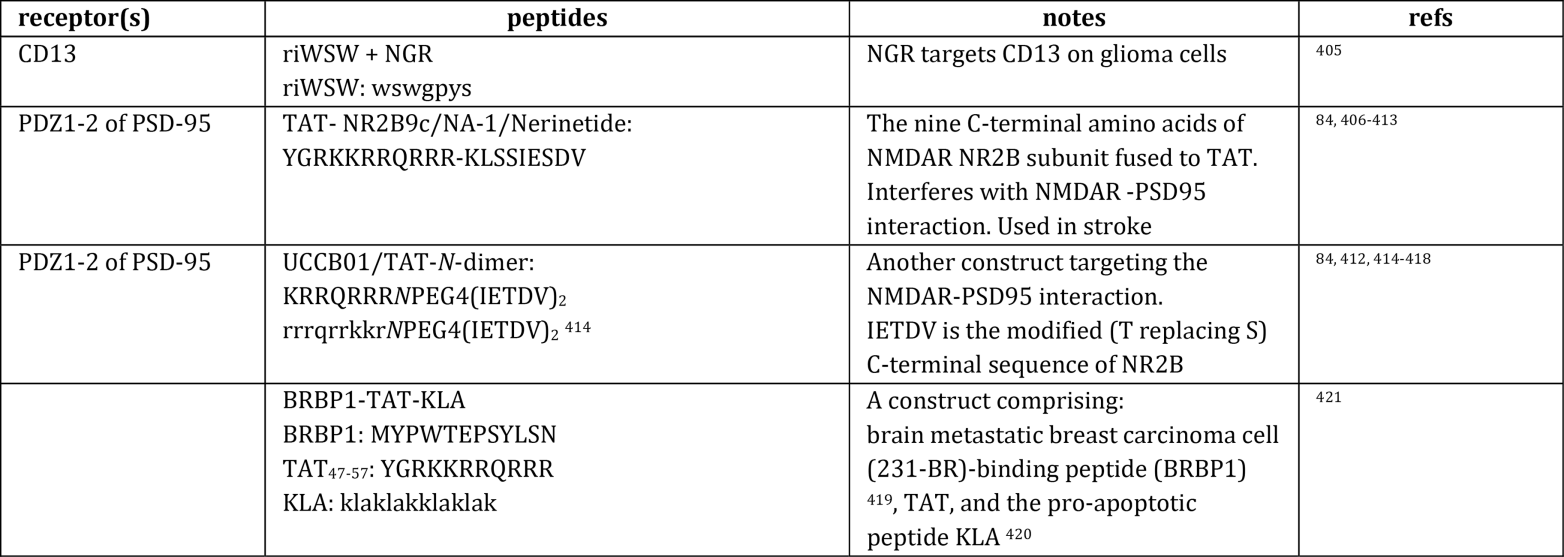
Multiple/Fusion Peptide Targeting^83,84,101,129,136,140,157,159,175,187,210,253,261,264,314,324,360−367,374−421^

a This table includes both fusion peptides and nanovehicles decorated with mulptiple peptides.

Singh and collaborators for example delivered genetic material or chemotherapeutics using liposomes carrying transferrin (Tf) and a CPP. As CPPs, the group used penetratin,^324,360−363^ vascular endothelial-cadherin-derived peptide (pVEC),^314^ pentapeptide QL,^314,364^ TAT,^314,324,364^ melittin (a component of bee venom),^365^ kFGF (a sequence from Kaposi fibroblast growth factor),^365^ Pas (a hybrid peptide joining a penetration accelerating sequence (Pas) and octa-arginine (R8),^365^ PFVYLI (derived from α1-antitrypsin),^366,367^ R9F2 (nona-arginine plus 2 phenylalanines at the C terminus),^367^ rabies virus glycoprotein-derived peptide (RVG peptide; this peptide actually targets AchR).^138−140^ This strategy proved remarkably successful, allowing brain delivery of 2.8% of the injected dose of Tf-kFGF liposomes,^314^ 3.4% of Tf-RVG,^140^ and 3.8% of Tf-TAT liposomes^314^ (assuming a 0.5 g mouse brain). Other studies with combinations of an addressing peptide and a CPP are tabulated in Table 4.

The efficacy of peptide-decorated nanoparticles (NPs) as mediators of brain delivery is thus often superior to that of NPs not labeled with peptides. The latter in some cases deliver to the brain only a minute fraction of the injected dose (e.g., refs (368) and (369)). For example, in the study by Calvo et al.,^368^ approximately 0.01–0.02% of the injected dose of ^14^C-labeled NPs entered the brain (at 30 min postinjection; assuming a 2 g rat brain). In the study by Brigger et al.,^369^ the fraction of i.v. injected ^14^C-labeled nanospheres found in the brain of rats was in the range 0.01–0.06% (depending on the characteristics of the NPs; assuming a 2 g brain) in control animals (or in the contralateral hemisphere; in an orthotopically growing tumor it was however found to be as high as 0.25% per gram of tissue). In other cases however, NP-mediated brain delivery can reach up toward 0.5–0.6% of the administered dose (e.g., refs (370−373)).

## 4 Shortcomings

Only a minority of the innumerable papers in the literature report negative or toxic effects of peptides or peptide-comprising molecular constructs or nanovehicles. This no doubt reflects the good overall profile of these materials, which generally show satisfactory biocompatibility. It clearly emerges however that generalizations are dangerous, that peptides may have important undesirable side effects and, in particular, that peptide conjugates may have properties quite distinct from those of the peptide itself.

There have been reports of antigenicity (i.e., peptides can, unsurprisingly, cause an immune response when conjugated to macromolecules/nanovehicles or due to aggregation to form supramolecular structures of sorts). In this respect, the report by Wang et al. is noteworthy in that cyclic RGD peptides such as cRGDyK (e.g., refs (129) and (176)) displayed on liposomes or conjugated to PEG_3400_ can induce a strong hypersensitivity response when re-adminstered.^422^ The response involved mainly IgG and IgM antibodies, cytokine release, and complement activation and resulted in anaphylaxis. The peptide administered by itself did not induce any such response. Different mouse strains showed different sensitivities, and the incidence of anaphylaxis could be reduced by enlarging the cyclic peptide ring or modifying the liposome composition, but immunogenicity remained.^423^

When d-CDX, a peptide targeting AchR and capable of passing the BBB, was grafted onto DOX-loaded liposomes, it accelerated their clearance following IgM absorption and caused serious liver problems.^130^ A shortened version, D8, was similarly effective in targeting and had lower side effects.^130^

According to at least one paper, TAT-containing peptidic constructs caused a weakening of BBB tightness, increasing the entry into brain parenchyma of FITC-labeled dextrans.^84^

CPPs, arginine-rich peptides in particular, appear to be potentially toxic. Thus, in mice TAT was reported to have an LD_50_ of 27 mg/kg bw (17.3 μmol/kg), CTP one of 21 mg/kg bw (13.5 μmol/kg), R11 one of 16.5 mg/kg (9.5 μmol/kg)^424^ (i.v. administration). LD_50_’s were lowered to 19 and 13 mg/kg bw (11.5 μmol/kg and 13.5 μmol/kg), respectively, by conjugation of TAT and CTP with GABA.^424^

The overall safety of TAT-containing peptides TAT-NR2B9c (TAT-KLSSIESDV, also known as NA-1 or nerinetide; see Table 4) and TAT-N/*O*-dimer (TAT-N/*O*-PEG_4_-(IETDV)_2_, also known as UCCB01-144; see Table 2) was evaluated by Bach et al.^412^ These are constructs aimed at ameliorating the consequences of stroke by interfering with the interaction of NMDAR (NR2 subunit, of which they reproduce/imitate the C-terminal) with the tandem PDZ1-2 domains of the four PSD-95-like MAGUKs in neurons. They did significantly reduce the infarcted area, but TAT-NR2B9c actually worsened the survival score because of cardiac complications and strongly lowered the heart rate and blood pressure in healthy control mice. TAT-N-dimer produced comparable protective results at lower dosages and had a better cardiovascular safety profile.

In a study with cultured cells, Saar et al.^425^ compared the toxicity (at 10 μM) of penetratin_43–58_, TAT_48–60_, pVEC_615–632_ (vascular endothelial-cadherin-derived peptide), model amphipathic peptide (MAP: KLALKLALKALKAALKLA), and transportan 10 (TP10). All the peptides used in the study were modified with a C-terminal amide. MAP and TP10 (K-rich peptides) turned out to induce significant LDH leakage, which depended on the cell line, as also indicated by the other *in vitro* studies. In an analogous study, Kilk et al.^426^ found that TAT, MAP, and, especially, TP10 (used at 5 μM) impacted the intracellular metabolome. Penetratin and R9 (Arg nonamer) instead had a negligible impact.

Jones et al.^427^ working with cultured cells, reported EC_50_’s of 6, 10, 17, and >100 μM for rhodamine-labeled trasportan, polyArg (R11), Antennapedia, and TAT-derived peptides, respectively. These authors also present evidence that toxicity in their system depends on “cargo”. This is confirmed for example by El-Andaloussi et al.^428^ Using the peptide TP10 coupled to carboxyfluorescein, these latter authors observed that toxicity also depended on the position of attachment of cargo to the peptide chain. The hemolytic activity of a TP10 conjugate at relatively high concentrations was confirmed also in a study showing its ability to ferry vancomycin into the brain.^328^

Possibly the most significant source of toxicity is the hemolytic potential of some peptides or peptide-cargo constructs. Again, this essentially concerns cell-penetrating peptides and harks back to the action of antimicrobial peptides, which also often display hemolytic activity (see refs (429−433)). These are, generally speaking, α-helical in the vicinity of lipid membranes and have a high content of both positively charged and hydrophobic amino acids. Lytic activity can be strongly influenced by apparently minor changes in amino acid composition.^434^

A clear example of this type of “complication” is provided by TP10, a shortened variant^435^ of transportan, a man-made combination of the N-termini of galanin (a porcine neuropeptide) and mastoparan (a wasp venom pore forming peptide).^436^ TP10 can act as a trans-BBB carrier (see Table 3). It has antiplasmodial (i.e. antimalaria) activity.^437^ Chloroquine and primaquine conjugates performed better than the unmodified drugs in this respect, but were strongly hemolytic.^438^ Analogous results have been reported for the conjugates of TP10 with ciprofloxacin or levofloxacin , two fluoroquinolone antibacterials.^439^ The peptide itself can form pores in lipid membranes^440,441^ and is hemolytic at high concentrations,^439^ but the conjugates were more powerful in this respect.

Hemolytic activity was also observed in the study comparing the BBB-permeating abilities of liposomes decorated with peptides pVEC, TAT or the pentapeptide QLPVM, each in combination with transferrin to target BBB cells.^314^ Measurable hemolysis was observed even at the lowest concentrations tested (31 nM phospholipids).

In recent work we exploited Angiopep2 or TAT to deliver to the brain PAPTP, a promising inhibitor of mitochondrial voltage-gated potassium channel 1.3 (Kv1.3), which completely lacks the ability to cross the BBB. Both Angiopep2 and TAT allowed the brain delivery of PAPTP (0.1% of the injected dose). However, the severe toxicity observed in the case of TAT-PAPTP forced us to focus the study on Angiopep2-PAPTP. TAT-PAPTP toxicity may be attributed, at least in part, to its hemolytic action.^115^

The ability to cause lysis can be put to good use, at least in principle, not only against noxious microorganisms but also against cancer, since cancer cells appear to be more sensitive to them than normal ones, probably due to differences in lipid composition.^442,443^ For example, breast and prostate cancers and their metastases have been attacked with lytic peptides conjugated to ligands of hormone receptors.^444−446^ Kawakami’s group has coupled, via a glycine triplet, a lytic peptide (KLLLKLLKKLLKLLKKK or KLlLKlLkkLLKlLKKK) with peptides targeting the epidermal growth factor receptor (EGFR),^447^ the transferrin receptor (TfR),^448^ the receptor for IL-4 (IL-4R),^449^ the epidermal growth factor receptor 2 (HER2/Erb2),^450^ and interleukin-13 receptor alpha 2 (IL-13Rα2)^451^ to obtain selective cancer cell-killing tools.

## 5 Challenges

The widespread application of peptides in the clinic is still hindered by a series of difficulties, summarized by ref (452). A major problem, at least for peptides composed wholly by natural amino acids, is their short lifetime *in vivo*, due to the abundance of peptidases in the digestive system (which limits oral administration), blood, liver, the BBB,^453^ and other organs (for tabulations, see ref (454)). Clearly, since the cell-penetrating or target-recognizing ability depends on sequence integrity, a rapid degradation is expected to lead to a lower effectiveness. Thus, for example, the half-life in human serum of HAI/T7, a 7 amino acid peptide targeting the transferrin receptor (see Table 1) was around 5 min (but increased to more than 24 h if the proteolytic sites were protected by *N*-methylation at the most labile positions or if the retro-inverso peptide was assayed).^158^ Similar (or lower) estimates were obtained with ^125^I-labeled peptides pVEC (<3 min), SynB3 (5.5 min), and TAT_47–57_ (2.7 min) (for refs to the peptides see Table 1) in mouse serum.^297^ These sequences were more resistant in liver, kidney, or brain homogenates, in which their half-lives ranged from 5 to 68 min. However, the concentration of TP10 (see Table 3) in human serum was halved in 22 h, and that of TP10–2, which differs from TP10 by the substitution of a proline for an alanine, was halved in about 4 h.^297^ The survival of peptides in the face of protease attack can be heavily influenced by “details” such as apparently minor variations in the sequence, the attachment of cargo or labeling, and the species in which the test is carried out. For example, in human plasma, the *t*_1/2_ of TAT_47–57_, which has 6 trypsin cleavage sites, was pegged at 3.5 min in the study by Grunwald et al.^455^^111^In-DOTA-TAT_48–61_ (DOTA is a metal chelator) instead required about 9 h.^456^

The (partial) remedies adopted by researchers may have an impact on peptide functionality and often require painstaking elaboration. They include cyclization, which blocks exopeptidases (e.g.,^457^), and can utilize disulfide bonds or “head to tail” formation of an amide bond. As an alternative, researchers can use N-terminal acetylation and/or C-terminal amidation, or otherwise blocking a peptide terminus, or “stapling” (i.e., linking two positions in the peptide with a hydrocarbon or other chain).^458^ Glycosilation^459,460^ and *N*-methylation of some of the backbone nitrogens or arginine residues is another such strategy,^461,462^ as is “capping” of some side-chain hydroxyl groups.^463^ Backbone *N*-methylation also favors membrane permeation.^464^ Once the protease-sensitive sites in the peptide have been identified, they can be “reinforced” by substituting some of the amino acids so as to make the cut less likely. Pro and Trp, sterically impacting, can be effective.^465^ Stabilization may also be sought by the introduction of unnatural amino acids (or β-amino acids)^466^ at selected positions and even changing the type of linkage between amino acids.^467−469^ The most effective and used approach to stabilization may however be the construction of “enantio” peptides, composed of d-amino acids in the same H- to NH_2_-terminus sequence as in the parent compound. This may however result in a reduction of the activity. Retro-inverso peptides, also formed by the same d-amino acids, however joined in the reverse C-to-N-terminus order, may help in such cases. The substitution of d- for l-peptides may also be partial. The applications of this strategy are many. Examples are provided by Prades and colleagues for the 12-mer THR peptide targeting TfR,^100^ by Schorderet and colleagues for TAT,^470^ and by Wei and collegues for Angiopep-2.^185^ Willbold’s group has developed a family of all-d peptides directed against the formation of Aβ oligomers (D3 (rprtrlhthrnr), D3D3, and RD2), which resisted oral administration, had a half-life of up 60 h *in vivo*, and had a positive impact on cognition in a genetic mouse model of Alzheimer’s disease.^471,472^ Another possibility is shielding the peptide by large PEG molecules, either linked to the peptide itself or juxtaposed on the surface of nanovehicles.^473^ The various approaches can be used in combination so as to optimize stability without interfering with selectivity and performance (for comprehensive overviews, see refs (474) and (475)).

A problem affecting many peptide-based delivery systems, especially those exploiting membrane receptors and nanovehicles, is that the construct may end up in the endosomal/lysosomal degradation pathway and be lost. Hence efforts to devise ways to promote the escape of the cargo from the endosome.^282,476,477^ Strategies are often based on the acidity of the endosomal compartment. The cargo may be linked to the peptide via an acid-labile group,^478^ or appropriate environment-sensitive “adaptor” peptides may be used.^479^ Engineered pH-sensitive vehicles may permeabilize or fuse with the organellar membrane under these conditions, releasing the cargo to the cytosol.^480^ Escape may for example be promoted by a fusogenic peptide such as H5WYG (GLFHAIAHFIHGGWHGLIHGWYG), derived from the N-terminal sequence of the HA-2 subunit of influenza virus hemagglutinin.^481^ Viruses have in fact achieved a high level of proficiency in endosome escape.^482^

In the specific case of trans-BBB delivery, the matter may be construed as the need to maximize transcytosis vs lysosomal degradation. Some attention has been devoted to this aspect in studies of oral/intestinal uptake, but more needs to be done, especially in the field of brain delivery. Ju et al. have recently reported some success in this direction by using a two-punch strategy.^483^ They relied on a previously developed “transcytosis targeting” peptide (TPP: LRQRRRLYC in their case) which binds to heparin sulfate.^484^ Nanovehicles decorated with this peptide are then endocytosed via lipid-raft-mediated endocytosis and are transcytosed. Ju et al. first treated their cells and mice with TPP-carrying NPs loaded with tunicamycin, believed to be an inhibitor of Mfsd2a (see above, section 2.4), then administered analogous NPs loaded with doxorubicin or a fluorescent marker. The “priming” procedure resulted in an approximately 4-fold increase in trans-BBB delivery.^483^

Empirically, the question of which BBB membrane receptor is engaged is relevant. LRP1, the receptor for An2, seems to perform better than TfR in this respect.^193^ Guo et al. reported using statins-loaded, Angiopep-2-decorated NPs to achieve upregulation of the expression of LRP1 in reaction to lowered cholesterol. This in turn resulted in reinforcement of subsequent transcytosis and drug delivery to brain metastases by LRP1-targeting An2-NPs.^193^

While there is little doubt that some peptides (e.g., TAT and Angiopep-2) can dramatically improve brain delivery of a “cargo” in comparison with its administration as such, in most cases this improvement still falls short of what a pharmacologist might desire. In other words, the efficiency of brain delivery often remains, in absolute terms, rather low. Examples follow.

The delivery of UCCB01-144 (TAT-N-PEG_4_-(IETDV)_2_)^416^ and UCCB01-125 (PEG4-(IETAV)_2_)^485^ to the brain was studied by Andreasen and collaborators. The molecules, whose purpose is to interfere with the interaction between the NMDA receptor and PSD-95, were labeled with 5-carboxyfluorescein and a 30 mg/kg bw (8.23 or 17.42 μmol/kg bw, respectively) dose was administered intraperitoneally to mice weighing approximately 23 g (range 20–26 g). The authors found 865 ± 113 and 107 ± 42 nmol/kg brain tissue, respectively, after 30 min from injection. Assuming a 0.5 g average brain, this translates to approximately 0.23% (UCCB01-144) and 0.013% (UCCB01-125) of the administered dose, respectively. The free (i.e., unbound) concentrations were calculated from equilibrium dialysis data to be on the order of 122 ± 16 and 10 ± 4 nmol/kg, respectively. The comparison between the TAT-comprising compound (UCCB01-144) and the TAT-less one (UCCB-125) highlights the usefulness of TAT as a brain-delivering device, but still one may note that the concentration of UCCB01-144 reached in brain (865 nmol/kg) was only about one-tenth of the concentration that would have been obtained if the drug had diffused evenly throughout the body of the animal (8230 nmol/kg).

The same group was i.v. injected with 7.5 mg/kg bw (equivalent to 567 nmoles per average animal) of carboxyfluorescein-labeled UCCB01-144 into rats with a mean bw of 251 g.^486^ The maximal concentration in the brain was 0.398 ± 0.123 nmol/g (at 1 h post injection). Assuming a 2 g brain, this translates to approximately 0.14% of the administered dose. In turn, since the unbound fraction was estimated at 11.5%, this corresponds to approximately 1.2% of the dose if the bound fraction is included.

In a recent study Kristensen and co-workers^84^ evaluated the delivery to brain parenchyma of carboxytetramethylrhodamine (TAMRA)-labeled peptides TAT, TAT-NR2B9c and TAT-*N*-dimer/UCCB01-144 (see above and Table 4) in mice. The animals received 3 nmol/g bw of the compounds via i.v. injection, and delivery was assessed by two-photon fluorescence microscopy of the brain as well as by extraction and fluorescence measurements of the lysates of various organs, including the brain. Fluorescence accumulated mainly in the kidneys, liver, and intestine but was excluded from the heart. At 1 h after injection, the intensity measured in the brain, including microvessels, corresponded to about 0.2% of the injected amount, for all three constructs, in agreement with the results of Andreasen and colleagues with UCCB01-14. Entry into the brain parenchyma appeared to be lower and considerably hindered by the presence of the “cargo” attached to TAT (i.e., the NR2B9c peptide or the N-PEG_4_-(IETDV)_2_ moiety), confirming that each construct may constitute a case apart. Phenomena such as self-association to form supramolecular complexes, variations in the extent of charge shielding, differences in adhesion to macromolecules in solution or to surfaces, and differences in the rate of proteolytic degradation may all contribute to these “cargo effects”.

Turning to another popular brain-delivery peptide, Angiopep-2 (see Table 1), quantitative estimates of brain delivery have been carried out with conjugates of the peptide with chemotherapeutics paclitaxel (ANG1005), doxorubicin (ANG1007) and etoposide (ANG1009). The i.v. injection of 14 nmol/g bw of radiolabeled ANG1005 (42 nmol/g bw of conjugated paclitaxel, linked via ester bonds) into 20 g mice led to the presence, after 30 min, of 0.62 nmol/g (calculated from radioactivity measurements, without actual knowledge of the chemical identity of the emitting species) in the brain parenchyma.^109^ This amount corresponds to about 0.11% of the administered dose (assuming a 0.5 g average brain) and represents a 54-fold increase over the brain delivery achieved by administering the same molar amount of unconjugated paclitaxel. Similar experiments with ANG1007 and ANG1009 resulted in the delivery of about 0.08 and 0.17%, respectively, of the injected dose.^181^ Again, these amounts represent remarkable increases in comparison to the administration of equimolar amounts of the unconjugated drugs. As may have been expected on the basis of the enhanced permeability and retention (EPR) effect, the delivery to the tumor mass in an orthotopic model of U87 glioma was considerably higher for both doxorubicin and etoposide; their Angiopep-2 conjugates however maintained their advantage, reaching about 1.2% of the administered dose in the most favorable case (ANG1009). This well-known higher accessibility of tumors, coupled with the higher efficiency of the conjugate, may explain the positive impact of at least ANG1005 in *in vivo* brain tumor models^108^ and in limited clinical trials with humans.^112,487^

As a final example, Sakamoto and co-workers^209^ measured in mice the brain uptake of ^125^I-labeled L57, a peptide selected via phage display, and Angiopep-7, both recognizing LRP-1. At 1 h after i.v. injection, the radioactivity counts found in the brain corresponded to 0.042 ± 0.017 and 0.032 ± 0.020%, respectively, of the injected dose.

As far as one can tell from the sparse quantitative reports, in many cases the delivery effectiveness is similar if these peptides are used to ferry across the BBB drug-loaded nanovehicles rather than individual drug molecules. For example, TAT has been anchored to the surface of doxorubicin-loaded liposomes with the intent of increasing the delivery of the drug to the brain.^488^ Mice then received via i.v. delivery a dose of liposomes carrying 2.5 μg/g bw of doxorubicin. The peak concentration of doxorubicin in the brain (at 1 h postinjection) was approximately 0.45 μg/g. Assuming an average mouse weight of 20 g and an average brain weight of 0.5 g, this works out to the delivery of about 0.45% of injected doxorubicin to the brain.

The same group^310^ compared the ability of four peptides to drive coumarin 6-loaded liposomes to the brain. The peptides were TAT-derived AYGRKKRRQRRR (**1**), its scrambled control RKARYRGRKRQR (**2**), a sequence reported as AYGGQQGGQGGG but possibly containing some glutamic acid residues (**3**), and octa-arginine (**4**). Uptake into various organs was evaluated at 1, 4, and 12 h after tail vein injection of 100 ng/g bw (0.1 mg/kg) of coumarin 6 contained in the differently labeled liposomes. The highest concentrations of coumarin were observed at the 1 h time point. At that time, the amounts found in the brain parenchyma were close to 2 ng/g tissue (2.5–3 ng/g if capillary depletion was not performed) for peptides **1**, **2**, and **4** and to 1 ng/g tissue for peptide **3**. Assuming again 20 g mice and 0.5 g brains, for the three best-performing vehicles this translates to a delivery to the brain parenchyma of approximately 0.25% (0.3–0.4% considering the brain with its capillaries) of the administered dose. An even distribution of the drug would have led to concentrations of about 100 ng/g, an approximately 50-fold higher level.

In an analogous study employing solid–lipid nanoparticles loaded with docetaxel (DTX) and Angiopep-2 as the targeting peptide, after i.v. injection of 10 μg/g bw DTX, the peak concentration of DTX in the brain was measured at 4.13 μg/g, which corresponds to about 0.9% of the dose.^201^

We have already mentioned however that better performances can be had with pluri-functionalized nanoparticles carrying different types of peptides. Another exception to the norm of a relatively low efficiency in trans-BBB delivery may furthermore be provided by some opioid peptides, in particular the glycopeptide g7, derived from the glycopeptide MMP-2200 and ultimately from leu-enkephalin (e.g. refs (489) and (490)). This peptide enters cells by multiple mechanisms and may be considered to be receptor-independent.^340^ It was used to decorate poly(d,l-lactide-*co*-glycolide) (PLGA) NPs marked to reveal their presence as a fluorescent spot.^338,491,492^ Quantifying the effects of the cargo (loperamide, an analgesic) and by direct analysis of the NPs and their cargo in the brain, the authors concluded that up to 15% of the injected (i.v.) dose of g7-decorated nanoparticles reached the brain of rodents.^339,340^ This remarkable success has been attributed to the ability of the glycopeptides to assume a specific conformation favoring its interaction with the BBB and folding to form an amphipatic α-helix, coupled to an enhanced water solubility conferred by the attached sugar moiety.^225,340^ In fact it has been argued that the presence of a glycosidic moiety may be an often-useful feature helping peptides to pass the BBB.^460^

Positively charged peptides (CPPs) obviously tend to bind to negatively charged biomolecules and structures, such as albumin^493^ and glycosaminoglycans (e.g., heparan sulfate, hyaluronic acid;^287,494^ or blood cells (see above)). Other aspects aside, this may result in hindrance to diffusion,^494−497^ lowered availability, and even analytical difficulties for the researcher.^115^

## 6 Conclusions and Perspectives

Peptides are a marvelous resource, but not all that glitters is gold. Like anything else, they need to be handled with caution, and they are not yet the cure-all for delivery problems, or, more specifically, for trans-BBB delivery problems. In most studies providing this type of information, the amount reaching the brain remained below par, which cannot be considered a satisfactory state of affairs even though enough active principle may have reached the brain to have an impact on the CNS pathology under study. In our opinion, peptides remain however a key component of the so-far elusive solution of the brain delivery problem. The search for more efficient sequences, the use of “stabilized” and/or “decorated” (e.g., glycosylated) peptides, the further development of cleverly engineered nanovehicles, and the ongoing exploration of innovative delivery routes (e.g., the nose-to-brain pathway) offer the perspective of steady progress toward the eventual implementation of a peptide-based technology affording the needed concentration of the drug in brain parenchyma.
